# Barriers to Managing Fertility: Findings From the Understanding Fertility Management in Contemporary Australia Facebook Discussion Group

**DOI:** 10.2196/ijmr.4492

**Published:** 2016-02-15

**Authors:** Sara Holton, Heather Rowe, Maggie Kirkman, Lynne Jordan, Kathleen McNamee, Christine Bayly, John McBain, Vikki Sinnott, Jane Fisher

**Affiliations:** ^1^ Jean Hailes Research Unit School of Public Health and Preventive Medicine Monash University Melbourne Australia; ^2^ Family Planning Victoria Melbourne Australia; ^3^ Department of Obstetrics and Gynaecology Monash University Clayton Australia; ^4^ The Royal Women's Hospital Melbourne Australia; ^5^ Melbourne In Vitro Fertilization Melbourne Australia; ^6^ Victorian Department of Health and Human Services Melbourne Australia

**Keywords:** fertility management, Australia, Facebook, social media

## Abstract

**Background:**

As part of research investigating the complexities of managing fertility in Australia, public opinions about how Australians manage their fertility were sought from women and men.

**Objective:**

To identify public opinion about sexual and reproductive health in Australia.

**Methods:**

To ensure access to a diverse group of people throughout Australia, an online group was advertised and convened on Facebook from October through December 2013. In a closed-group moderated discussion, participants responded to questions about how people in Australia attempt to manage three aspects of fertility: avoiding pregnancy, achieving pregnancy, and difficulties conceiving. Nonidentifiable demographic information was sought; no personal accounts of fertility management were requested. The discussion transcript was analyzed thematically.

**Results:**

There were 61 female and 2 male Facebook users aged 18 to 50 years living in Australia participating in the study. Four main themes about fertility management were identified: access, geographical location, knowledge, and cost. Participants reported that young people and people from rural areas face barriers accessing contraception and fertility services. Limited knowledge about sex and reproduction and the cost of fertility services and contraception were also said to impede effective fertility management.

**Conclusions:**

Reasons for inequalities in effective fertility management that are amenable to change were identified. Facebook is an effective method for gaining insights into public opinion about sexual and reproductive health.

## Introduction

Avoiding pregnancy when it is not desired and achieving a desired pregnancy are matters of concern throughout the reproductive life course. Modern contraceptives and assisted reproductive technology (ART) have contributed to fertility management in what has been termed the “reproductive revolution” [1]. In contemporary high-income democracies such as Australia, these are presumed to enable all women and men to manage fertility optimally [2,3].

Australia faces the paradoxical problems of high rates of unintended pregnancy and of infertility, and many Australians do not achieve their reproductive preferences [4,5]. We know that most Australians want to have children [4,5]. Little is known about how contemporary Australians of reproductive age manage fertility [1,2].

Modern oral contraceptives first became available to married and later unmarried Australian women about 50 years ago and were thought to have initiated the era of the planned and wanted pregnancy and the end of the unplanned pregnancy. Subsequent surveys of contraceptive use, pregnancy intention, pregnancy outcome, and ideas about family formation have found that this has not been realized. For example, although the 2001-2002 Sex in Australia survey of a representative sample of 9134 people aged 16 to 59 years found that only 70% of sexually active women of reproductive age were using modern contraception [6], 22.6% reported a past induced abortion and 16.9% of women aged 20 to 29 had become pregnant during adolescence [7].

Infertility, the inability to conceive after 12 months of unprotected intercourse, occurs in 1 out of 6 Australian couples [8]. Even with ART, not all people who desire to conceive do so, even with repeated treatment cycles; success rates decline with maternal age. Financial expense, health problems, and the psychological costs of repeated experiences of hope and despair are, for some, barriers to continuing treatment [9,10], but most people appear to view these technologies as affordable, accessible, benign, and highly effective. For example, only one-third of secondary school students understand that ART does not cure infertility [11], and some adults in their mid-thirties delay conception because they believe ART is a reliable alternative if conception is difficult [12]. Most women and men who participated in the Australian Institute of Family Studies’ Fertility Decision-Making Project in 2004 believed that they were likely or very likely to succeed in having children through in vitro fertilization (IVF), and those in their late thirties were as likely as those in their twenties or early thirties to be optimistic about conceiving with IVF [13].

Social networking sites such as Facebook are extremely popular. Facebook is the largest and most widely used social networking site [14]; in January 2015, Facebook had almost 14 million users in Australia [15]. Research using social network sites is a relatively new phenomenon [16], but it has been viewed positively by research participants [17]. To date, health-related research using Facebook has mostly explored its use as a health resource or a way of recruiting study participants [18]. There has been limited use of Facebook as a platform for online discussion groups. Facebook provides valuable opportunities for researchers to engage people from diverse backgrounds and locations, including traditionally hard-to-reach groups such as younger people, in a space in which they are comfortable and open to discussing their ideas and opinions [14,19]. Social networking sites have the advantage of enabling participation in online discussion groups at times that suit participants [20], and they can be accessed via personal computers, smartphones, and tablets, removing the need for a physical venue [16].

The aim of this research was to investigate public opinion about fertility management in Australia. To ensure that participation was available as widely as possible throughout the country, an online discussion group was advertised and convened on Facebook.

## Methods

### Study Design

This study is part of a multimethods research project that includes a population-based survey and in-depth interviews.

### Sample

English-speaking women and men aged 18 to 50 years who were Facebook users living in Australia were sought and invited to participate in the online discussion group.

### Recruitment and Procedures

For the study, the researchers created a closed (private) group on Facebook. The member list of a closed group is visible to all Facebook users, but only members can read what is posted. Before the group began, researchers made decisions about reasonable expectations for privacy, ownership of any data generated, and means of moderating the discussion and removing any offensive posts. These expectations were outlined to potential participants on the project’s Facebook page.

**Figure 1:**
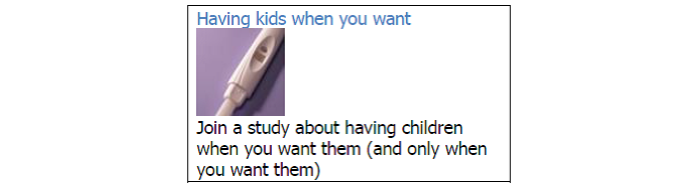
Facebook advertisement.

From October through December 2013, an advertisement (see Figure 1) briefly describing the research and discussion group was placed on the Facebook pages of all users meeting the eligibility criteria. Age was identified from the user’s profile (a mandatory field on all personal Facebook accounts), and location was established from the Internet protocol address [21]. We chose the option of being charged per click rather than per thousand impressions because we were seeking people who would click through to our Facebook project page from the advertisement [22].

The project page provided further details about the research and the participation involved. A participant information statement explained that the discussion would be about their perceptions of how people in Australia manage their fertility and there would be no personal questions about participants’ own fertility management. Those who chose to participate requested to join the group by clicking on a link on the Facebook project page. The group moderator (SH) approved requests and sent participants a “welcome to the group” message via Facebook inviting them to participate in the discussion by posting their responses to questions and to comments from other members.

To develop a summary description of group members, a link to an online survey was included in the welcome message to new participants. The survey, which was not part of the group discussion, contained questions about demographic variables (age, sex, relationship status, country of birth, level of education, Aboriginal or Torres Strait Islander status) and fertility history (past fertility problems or accidental pregnancy, number of children).

The research team developed a guide with the following questions to initiate and prompt discussion on community views and attitudes about avoiding and achieving pregnancy.

- What do you think people do (or don’t do) when they want to avoid a pregnancy?
- What services might be available to people who want to have sex but not get pregnant?
- What do you think people do (or don’t do) when they want to get pregnant?
- What services might be available to people who want to get pregnant?

Participant responses were read daily by the moderator and discussed at least weekly by the research team. The moderator asked additional or clarifying questions as appropriate and did not censor personal anecdotes. Members of the group also commented on each other’s posts. The moderator posted a new question every few days.

### Data Analysis

The transcript of the group discussion was copied from Facebook and pasted into a Word document. Participants were identified in analysis by number only. Data were analyzed using the four systematic steps appropriate for focus groups [23,24]: organizing, shaping, summarizing, and explaining. The first author systematically coded the transcript into emergent themes and subthemes. The transcript and themes were reviewed by all authors; discrepancies were discussed and agreement reached on the final themes.

### Ethics

The main ethical considerations were privacy and voluntary participation. Facebook allows users to determine how much of their personal information is publicly displayed. Profile security settings can be public (ie, allowing access to the complete profile by any Facebook user) or private (ie, limiting access of some or all profile information). Before joining the discussion group, participants were asked to ensure that their Facebook privacy settings were consistent with what they wanted to reveal to the group. Participation in the group was voluntary, and participants could withdraw at any time. A request to join the group was taken as informed consent to participate.

The research project was approved by the Human Research Ethics Committees of Monash Health (Project Number 11280B, September 2011), Monash University (Project Number CF12/0302-2012000125, February 2012), the Royal Women’s Hospital (Project Number 11/44, December 2012), Family Planning Victoria (Project Number 11/3, February 2012), and Melbourne IVF (Project Number 10/12, April 2012).

## Results

### Participants

The Facebook advertisement was viewed 60,372 times. There were 783 clicks on the advertisement (directing respondents to the study Facebook page) and 63 requests to join the group. All requests were accepted; 61 women and 2 men joined in the group. Comments were posted by 13 (21%) members of the group. The group ran for 3 months, from October through December 2013.

Demographic data were provided by 46 (73%) participants. It was not possible to distinguish the demographic information of contributing participants from noncontributing participants or associate demographic information with individual posts. The mean age of participants who provided demographic data was 30.1 years (18-49 years). One participant identified as Aboriginal or Torres Strait Islander. Participants were mostly born in Australia (38/46, 83%); the remainder were born in Canada, Indonesia, Malaysia, New Zealand, Sri Lanka, Ukraine, and the United States. Most participants (40/46, 87%) had completed postsecondary education and were married or living in a heterosexual relationship (37/46, 80%). In this study, 2 participants were living with a partner of the same sex; 5 were not currently in a sexual relationship.

The participants had varied fertility experiences. Approximately 40% (19/46) had experienced fertility difficulties, and a similar proportion (19/46, 41%) reported an accidental pregnancy. Just over half (24/46, 52%) had had children (ranging from 1 to 4).

### Key Themes

Guided by questions posted by the moderator, the discussion focused on 3 main aspects of fertility management: avoiding pregnancy, wanting to get pregnant, and difficulties getting pregnant. Analysis revealed 4 key factors identified by the participants as affecting an individual’s ability to manage her/his fertility effectively: access to contraception, geographical location, level of knowledge about sex and reproduction, and the cost of fertility services and contraception.

#### Theme 1: Access to Contraception

Contraception was identified as the main means of avoiding an unwanted pregnancy. Participants discussed various contraceptive methods: the oral contraceptive pill, long-acting hormonal contraception for women (such as hormonal intrauterine devices), condoms, ovulation calendars, testing of cervical mucus, and herbal preparations. The oral contraceptive pill was regarded as the preferred and most commonly used contraceptive method. However, participant responses indicated that choice of contraceptive varied among age groups. Teenagers were thought to use condoms more because they are easy to access.

A younger teen is more likely to use condoms (if they use anything) because you don't need an appointment with a doctor to get condoms.

Participant #1

For the youth it also requires considerable planning to obtain a script for the pill or implant et cetera.

Participant #2

Younger women were thought to prefer an implant or oral contraceptive pill and use emergency contraception frequently. In contrast, older women who are in committed relationships and no longer wanted to get pregnant were viewed as preferring surgical methods because these are permanent and do not require management.

I live in a larger regional center and find that many women in ongoing relationships that are over 30-35 [years] prefer the male partner to have a vasectomy. Then if he won't she tends to have the tubal ligation.

Participant #2

Participants commented that ideally both partners in a relationship should be actively involved in making decisions about the use of contraception and avoiding unwanted pregnancies. However, participants expressed the view that, among younger people, there was a perception that women should manage contraception, and some men refuse to use contraception.

A lack of communication between partners was also perceived to influence contraceptive use, especially for younger people.

I think there is unfortunately still an awkwardness (particularly in young people) about actively talking with partners about contraception. The maturity to physically have sex doesn't always come with maturity to really think about the consequences that come with that responsibility or to have open conversations with partners to actively plan for and manage contraception choices. The “it won't happen to me” attitude and poor communication skills can lead to complacency and risk taking.

Participant #3

Participants also commented that health care professionals rarely discussed sex or sexuality with young people and recommended more education for health care professionals and parents about how to talk to young people about their sexuality and sexual health. Social media was identified as an appropriate and effective platform for facilitating discussions between health professionals and young people about contraception and fertility management

What would help [people manage their fertility]? GPs, MCHNs, social workers spending more time explaining contraception to young people and debunking the myths. Increased education for health professionals and the general public.

Participant #4

#### Theme 2: Geographical location

Frequent reference was made to the barriers limiting access to fertility services including more permanent contraceptive methods and abortions, particularly in rural areas.

It often takes 4 weeks just to get a GP appointment in the country/regional areas.

Participant #2

There are many options open for city people not so many for country folks however.

Participant #5

I live in a rural city and it is virtually impossible for a woman to get a tubal ligation here. The Ob-gyns just won't do it.

Participant #1

I also live in a larger regional center but not many women here get tubal ligations as there are only 2 Ob-gyns here and neither are keen on surgical intervention for contraception for women. There is one surgeon who will do vasectomies.

Participant #2

There is no one in my large regional center that will do a termination. Women are forced to travel at least 2 hours to access legal abortion. And being that the clinics won’t allow you to leave without someone with you.

Participant #1

Participants also discussed the impact of location and income on access to fertility services for overcoming conception difficulties. ART was identified as a last resort after all of the other options are exhausted, but participants asserted that there should be universal access, lamenting the limited options for people living in rural areas or with inadequate financial resources.

We live in a rural area, though are very fortunate to have a fantastic reproductive medicine clinic for our region. I am not sure this would be so easily accessible if you didn't live in easy travel distance of such a specialist. So I guess to answer further, while getting the referral and actually accessing this clinic might be easily done, I think the reality is not so straightforward.

Participant #3

Yes of course [everyone should have access to reproductive services]!!!! Not everyone wants or can live in a big city!!!...I understand that it is cost-prohibitive to have a permanent IVF facility in country hospitals/health centers!!! But what about a traveling team!!! Surely that would be possible!

Participant #5

#### Theme 3: Level of Knowledge About Sex and Reproduction

Participants reported that although many people do not want a pregnancy, they often do not use contraception or use it incorrectly. This may reflect, at least in part, their level of knowledge about sex and reproduction. School-based sex education was identified as an inadequate and often inaccurate source of information, and sometimes it was absent altogether. Many people relied on their peers or the Internet for information about contraception.

I went to a girl’s school and some of the...girls had no idea!! They honestly thought that if you smoked alpine [cigarettes]...you didn’t get pregnant!!! We soon informed them differently!!!

Participant #5

One of the nuns told us that if we to go out with a boy—take a phone book to sit on (if we are going to sit on his lap—to prevent sperm getting to us because boys leak).

Participant #2

[School] didn't teach me anything to do with sex ed or avoiding a pregnancy. My mother had the conversation with me and I looked up information on the Internet.

Participant #6

I've found loads of things in Google.

Participant #7

I’ve found young people often find it the most difficult to determine whether info is reliable on the Internet. Some...then contact friends on Facebook to ask what they think or what their experience is. In comparison, someone like myself may consult government health websites or community organizations and then clarify this with a medical professional in person.

Participant #4

Participants thought that a lack of education about contraception and reproduction also made it difficult for disadvantaged or young people to realize their intentions and to avoid unwanted pregnancies.

Individuals from disadvantaged backgrounds may be more likely to opt for using condoms or the pill because they are uneducated about the other options available. Condoms are also easier to access from service stations et cetera whereas getting a script for the pill or other types of contraception involves attending a GP or clinic. Many young people were unaware they could drop in to local family planning clinics for free.

Participant #4

Young women rely on peers for information about contraception. I have worked with many young women over the past three years who had unplanned pregnancies, many who had more than one unplanned pregnancy and did not know the variety of contraception available, as well as the low cost if you have a health care card.

Participant #4

Participants also discussed the impact of a person’s level of knowledge about sex and reproduction on achieving a wanted pregnancy. They identified the main actions to be adopted as increasing the frequency of sexual intercourse and having sex at the “right time.” Participants also advocated monitoring ovulation and knowing “how our bodies work” as contributors to achieving conception.

A few months in, I started tracking my ovulation, having sex at the right time.

Participant #8

My husband works away for 9 days and home for 5 days. I used a period tracker phone app to monitor my cycles and get an idea when I would ovulate. Then we had sex every 2nd day as I have read that's best for trying to conceive.I also started to track cervical mucus and attempted to chart temp but wasn't accurate. I personally have looked into all the ways to determine ovulation to make it easier to conceive.

Participant #9

Changes to practices and behavior, including improving diet, ceasing smoking, and limiting alcohol and drug use were also recommended by the group for achieving conception.

Personally, trying to get pregnant was a bit of a staged process. I was so excited about starting to try that I cut down alcohol, had been taking multivitamins and folate for months before, said no to events “just in case.”

Participant #8

Lack of recognition of men’s roles and responsibilities in achieving a pregnancy were also identified.

I think women so often “cop the flack” for fertility issues, but it disregards the role of men's health and fertility which is equally important. You seldom hear of men quitting drinking or getting healthier in order to conceive yet they contribute half of the DNA to the process! I think definitely both partners need to be actively involved and responsible.

Participant #3

It is seen as the woman [who] needs to get healthy to carry the baby...it seems to be forgotten that first both parties have to create the baby!!!

Participant #10

It was claimed that men are neither very aware of or concerned about women’s age-related fertility decline.

I don't think it’s that easy to get pregnant, especially as you start to reach your thirties. This is something that I and my girlfriends are concerned about. Our husbands however don't seem to understand what all the fuss is about. There's been a lot more focus in the media in the last few years regarding the risks of having children later.

Participant #4

Despite participants’ perception that health care professionals lacked knowledge about contraceptive options, a range of health services, such as reproductive medicine clinics and naturopaths, was identified as important sources of information about achieving pregnancy. General practitioners (GPs) and the Internet were regarded as the main sources of information.

If I was planning to [get pregnant] I would probably access my GP as a starting point. I'd also probably jump online to find out information before even going to the GP.

Participant #4

Participants commented that they had assumed it would be easy to conceive because it had been so for their friends and family but found that their own experiences challenged this assumption.

I am one of those that thought I would just stop the pill and instantly become pregnant, why wouldn't I, the rest of my family did!...I was super naïve...I have a number of girlfriends who literally pick a month and without understanding when ovulation occurs achieve it exactly as planned!

Participant #10

We expect that [pregnancy] will happen when we want it to and cease contraception.

Participant #3

The emphasis in sex education on avoiding pregnancy was also cited as contributing to the common belief that it is easy to get pregnant.

Based on my conversations with my daughter (15 year old), [people learn at school] absolutely nothing. I think in my region the myth that getting pregnant is the easiest thing in the world for everyone (and one I fell for in my younger days) is being furthered by the lack of conversation and education.

Participant #2

#### Theme 4: Cost of Fertility Services and Contraception

The cost of fertility services and contraception was also identified as a barrier to effective management of fertility.

We are potentially staring [down] the barrel of more IVF and it’s so expensive, and last time we did it was just after funding from IVF was redirected to lap band surgery.

Participant #10

Many people would lack the financial capacity to actually afford to access these specialist [reproductive medical] services, and if they can to begin with, repeated treatments and the associated expenses can very quickly drain the average budget.

Participant #3

The costs of even condoms could be prohibitive for some people. I don't think many people are aware of the mechanisms to obtain free condoms from planning clinics or community health centers. If people are needing to prioritize food with contraception, there is likely to be that inclination to overlook it as a priority regardless of the consequences.

Participant #3

With the defunding of many community-based programs, no doctors [are] left that bulk bill et cetera. The reliance on condoms and the morning after pill seems to be increasing.

Participant #2

## Discussion

### Principal Findings

This study identified key themes in the understanding of fertility management in contemporary Australia. Despite the participants having a variety of views and fertility experiences, similar and consistent barriers to effective fertility management were identified: avoiding or achieving pregnancy and conceiving when faced with fertility difficulties. Younger people, people living in rural areas, and people with lower levels of knowledge about sex and reproduction were thought to have the most difficulties in managing fertility.

This study was original in its innovative use of Facebook as the platform for an online discussion group. Facebook was a cost-effective and efficient way of enabling people from diverse geographical locations to participate in the group discussion at times that were convenient for them. As a result, this study included a diverse sample of women and men living in metropolitan and regional areas of Australia with varied fertility experiences.

### Limitations

The primary limitation of the study is the self-selection of the sample. The study was advertised as being about fertility management, and almost half of the participants had experienced fertility difficulties. Only 2 were male—more targeted approaches may be required to encourage men to participate in research to assess community views about sex and reproduction.

We found, as have others [25], that contraceptive practice varies by age; younger women are more likely to use oral contraceptives or condoms while older women are more likely to use permanent methods. These results may reflect childbearing patterns and sexual practices of younger people, who may be more likely to have changing sexual partners and need protection from sexually transmissible infections [26]. Participants also commented that access to long-acting and permanent methods of contraception was more difficult for people living in rural locations. Our findings from the survey component of this research indicate that lack of ready access to preferred contraceptive methods may constitute a barrier to fertility management for people living in rural areas [27]. This may reflect difficulties in rural areas of accessing health services and health professionals trained in insertion of long-acting reversible contraception.

A key finding was the perception that people’s level of knowledge about sex and reproduction is inaccurate and inadequate for their needs. This confirms existing evidence of considerable knowledge gaps about avoiding and achieving pregnancy, including low awareness of when women are most fertile [28]; naivety about the likelihood of experiencing fertility difficulties [29]; and the inadequacy of school-based sex education in topics such as emergency contraception, fertility, and pregnancy [30].

Costs of fertility services and contraception were also identified as barriers to effective fertility management. Raising awareness of how and where to access affordable contraception will allow people to use effective contraceptives that suit their needs. Many effective methods (eg, oral contraceptive pills, implants, and hormonal intrauterine devices) are subsidized through the Pharmaceutical Benefits Scheme. However, Sexual Health and Family Planning Australia [31] has argued that the out-of-pocket costs of long-acting reversible contraception may be unaffordable for many women. These findings corroborate those of the survey component of this research which also showed that relative social disadvantage was associated with significantly increased odds of unintended pregnancy [27].

### Policy and Practice Implications of Findings

The identified barriers to fertility management are all potentially modifiable. Education and public awareness campaigns about sex, reproduction, and available community health services should be undertaken and especially directed at young people. Knowledge gaps could also be alleviated by giving primary care providers (and others including social workers, teachers, and youth workers) adequate training for communicating about sexual and reproductive health (eg, taking time to explain contraception to young people and debunking common misconceptions). Government-sponsored public awareness campaigns were also viewed as important in educating people about the significance of men’s role in fertility management. Traveling teams of sexual and reproductive health providers could increase access to essential services in rural areas.

### Conclusion

The results of this study reveal community awareness of modifiable factors that contribute to sexual and reproductive health inequalities in Australia and demonstrate the effectiveness of using social networking sites such as Facebook for sensitive health-related research.

## Abbreviations

ART: assisted reproductive technology
GP: general practitioner
IVF: in vitro fertilization
